# Editorial: Extracellular Vesicle Treatment, Epigenetic Modification and Cell Reprogramming to Promote Bone and Cartilage Regeneration

**DOI:** 10.3389/fbioe.2021.678014

**Published:** 2021-04-21

**Authors:** Yixuan Amy Pei, Yufeng Dong, Tong-Chuan He, Wan-Ju Li, Wei Seong Toh, Ming Pei

**Affiliations:** ^1^Stem Cell and Tissue Engineering Laboratory, Department of Orthopaedics, West Virginia University, Morgantown, WV, United States; ^2^Department of Orthopedic Surgery, Center for Tissue Engineering and Regenerative Medicine, Louisiana State University Health Sciences Center, Shreveport, LA, United States; ^3^Molecular Oncology Laboratory, Department of Orthopaedic Surgery and Rehabilitation Medicine, The University of Chicago Medical Center, Chicago, IL, United States; ^4^Department of Orthopedics and Rehabilitation, University of Wisconsin-Madison, Madison, WI, United States; ^5^Faculty of Dentistry, National University of Singapore, Singapore, Singapore; ^6^Department of Orthopaedic Surgery, Yong Loo Lin School of Medicine, National University of Singapore, Singapore, Singapore

**Keywords:** extracellular vesicle, decellularized extracellular matrix, cell reprogramming, bone regeneration, cartilage regeneration, cell sheet, tissue engineering

Trauma, tumor, and age-related degeneration can damage bone and cartilage beyond a critical point, resulting in bone loss, cartilage defects, and osteoarthritis if left untreated. Traditional bone transplantation relies on autografts and allografts, which are limited in availability and have poor clinical efficacy due to donor site morbidity, immune issues, disease transmission, and unpredictable autologous resorption (Betz, 2002; Benic and Hämmerle, 2014). Similarly, cartilage restoration techniques like autologous chondrocyte implantation, osteochondral transplantation, and microfracture often lead to fibrotic scar tissue formation, which can cause pain and which have inconsistent long-term results (Valderrabano et al., 2009; Levy et al., 2013). Recent advances have recognized innovative approaches in enhancing bone and cartilage regeneration after injury. The wide array of treatment options used to support the body's regenerative abilities ranges from scaffold enhancement (Qasim et al., 2019) to extracellular vesicle (EV)-based therapies (Toh et al., 2014; Negoro et al., 2018).

Although cell sheet technology has been widely used in tissue engineering (Imashiro and Shimizu, 2021), Xu et al., found that *in vitro* high-density cultures trigger human bone marrow-derived mesenchymal stem cells (BMSCs) to undergo spontaneous cell senescence with a gradually increasing inflammatory profile and a build-up of reactive oxygen species. This finding is in line with a previous report, in which human synovium-derived stem cells (SDSCs), which contributed to the deposition of extracellular matrix (cell sheet), exhibited a significantly decreased chondrogenic potential (Zhang et al., 2015).  Xu et al., also found that this effect was reduced in Wnt3a-treated cells, as Wnt signaling is thought to activate cell cycle inhibitor p27, redirecting cells into quiescence instead of senescence, which provides a solution to generating multiple layer cell sheets with mitigated cell senescence.

Bone substitutes, which are engineered and implanted into the body to support tissue remodeling, are popular alternatives to autografts and allografts. This regenerative technique replicates the natural bone microenvironment by combining biomaterial scaffolds with growth factors and progenitor cells, thus providing a site onto which cells can attach, spread, migrate, proliferate, and differentiate. Zhang et al., reviewed various scaffold biomaterials, including inorganic compound-based ceramics, organic natural polymers, and synthetic polymers, which can be used in bone tissue engineering. Depending on the composition, biomaterials vary in effectiveness across the categories of biocompatibility, biodegradability, osteoconductivity, osteoinductivity, and mechanical properties. Interestingly, tissue-derived decellularized extracellular matrix (T-dECM) attracted more attention due to its mimicking of the complex microenvironment of native bone tissues.

Compared to bone, cartilage is known to be more difficult to regenerate due to its avascular nature, low chondrocyte proliferative ability, low progenitor cell count, and slow matrix turnover. Zhao et al., summarized the different natural and synthetic polymer scaffold biomaterials used in cartilage engineering. Along with the use of T-dECM, scaffold-based 3D bioprinting techniques including laser technology, extrusion, and jetting, are capable of mimicking the native tissue environment by delivering live cells and their corresponding scaffolding material in a precise and customizable manner. T-dECM has been recognized as a natural scaffold for engineering tissue; intriguingly, cell-derived dECM (C-dECM) acts differently by providing an *in vitro* microenvironment for the rejuvenation of primary cells and MSCs in proliferation and differentiation capacity for cartilage regeneration (Sun et al., 2018).

Outside of these commonly used biomaterials, a new composite scaffold, tannin, has been proposed by Yang and Abdalla. Resembling ceramic, porous tannin spray-dried powder (PTSDP) has considerable potential for use in bone graft engineering. They found that the macroporosity of tannin enhanced scaffolds was maintained by controlling the number and diameter of polyethylene glycol particles, thus enhancing infiltration, communication, and growth of human induced pluripotent stem cell-derived mesenchymal progenitors to better mimic the bone microenvironment. The study also found that these PTSDP scaffolds support long-term viability, attachment, and osteogenic differentiation.

Besides scaffold enhancement, there has also been increasing interest in incorporating cellular components with endogenous therapeutic capability into tissue regeneration. Hypoxia inducible factor-1α (HIF-1α) is known to be integral to bone defect repair, but it is typically unstable under normoxic conditions. Ying et al., found that mutant HIF-1α (a stabilized version of HIF-1α) stimulated rat BMSCs' proliferation and osteogenic differentiation in culture. Furthermore, *in vivo* studies demonstrated that mutant HIF-1α applied to a porous β-TCP scaffold promoted bone regeneration and neovascularization in mouse cranial defects 12 weeks post-surgery.

There is increasing evidence supporting the use of EVs from MSCs as a therapeutic device, as the wide array of components such as mRNA, miRNA, lipids, and bioactive proteins within the vesicles serve as mediators of intercellular communication and can promote wide-ranging tissue repair and regeneration, including cartilage repair (Toh et al., 2017). To et al., conducted a systematic review of 10 case-control *in vivo* studies looking at 159 murine subjects and found that the surgical application of human MSC-derived EVs reduced cartilage loss in cartilage injury sites. These EVs represent a cell-free option for cartilage repair that avoids the many risks of cell-based therapies, such as metastasis. Cumulatively, these studies indicate that EVs are capable of impacting various signaling pathways in many different cell types, not only directly promoting chondrogenesis but also decreasing macrophage-induced inflammation to support cartilage repair. Given that these studies use different EV isolation methods, animal models, and dosing regimens, future studies with better standardization are required.

As the authors explore new avenues of skeletal tissue regeneration, the future of tissue bioengineering can be seen heading in the direction of personalized medicine. Scaffold enhancement has become a widespread method to incorporate different factors that can more fully mimic the physiological function and architecture of bone tissue. Given the promise of EVs in therapeutic applications, it is important to recognize the epigenetic roots that underlie bone and cartilage degeneration (Van Meurs et al., 2019) and the potential use of chemicals and pharmaceuticals to promote the desired epigenetic modifications that drive chondrogenic and osteogenic differentiation in scaffolds (Eslaminejad et al., 2013). Indeed, EVs have potential to serve as vehicles for drug and molecule delivery to support skeletal tissue regeneration. However, despite the therapeutic potential of these new therapies, we must be cautious of the uncertain impact that these foreign substances may have on endogenous signaling pathways and the long-term robustness of the repair tissue. Any safety risks associated with these new technologies must also be fully scrutinized before they are translated into clinical applications.

## Author Contributions

YAP performed the collection and assembly of data, data analysis and interpretation, manuscript writing, and final approval. YD, TCH, W-JL, and WST performed data analysis and interpretation as well as final approval. MP performed conception and design, data analysis and interpretation, manuscript writing and final approval, and financial support. All authors contributed to the article and approved the submitted version.

## Conflict of Interest

The authors declare that the research was conducted in the absence of any commercial or financial relationships that could be construed as a potential conflict of interest.
